# The Effect of Biomolecular Gradients on Mesenchymal Stem Cell Chondrogenesis under Shear Stress

**DOI:** 10.3390/mi6030330

**Published:** 2015-03-02

**Authors:** Alexander L. Rivera, Harihara Baskaran

**Affiliations:** 1 Department of Biomedical Engineering, Case Western Reserve University, Cleveland, OH 44106, USA; 2 Department of Chemical and Biomolecular Engineering, Case Western Reserve University, Cleveland, OH 44106, USA

**Keywords:** tissue engineering, mesenchymal stem cells, chondrogenesis, microfluidic devices, cartilage, TGF-β1 gradients, shear stress

## Abstract

Tissue engineering is viewed as a promising option for long-term repair of cartilage lesions, but current engineered cartilage constructs fail to match the mechanical properties of native tissue. The extracellular matrix of adult human articular cartilage contains highly organized collagen fibrils that enhance the mechanical properties of the tissue. Unlike articular cartilage, mesenchymal stem cell (MSC) based tissue engineered cartilage constructs lack this oriented microstructure and therefore display much lower mechanical strength. The goal of this study was to investigate the effect of biomolecular gradients and shear stress on MSCs undergoing chondrogenesis within a microfluidic device. Via poly(dimethyl siloxane) soft-lithography, microfluidic devices containing a gradient generator were created. Human MSCs were seeded within these chambers and exposed to flow-based transforming growth factor β1 (TGF-β1) gradients. When the MSCs were both confluent and exposed to shear stress, the cells aligned along the flow direction. Exposure to TGF-β1 gradients led to chondrogenesis of MSCs, indicated by positive type II collagen staining. These results, together with a previous study that showed that aligned MSCs produce aligned collagen, suggest that oriented cartilage tissue structures with superior mechanical properties can be obtained by aligning MSCs along the flow direction and exposing MSCs to chondrogenic gradients.

## 1. Introduction

Osteoarthritis, characterized by the degeneration of cartilage that lines the articulating surfaces of bones, is a debilitating condition that affects over 48 million individuals in the United States alone [1,2]. Articular cartilage is an avascular tissue with a poor intrinsic healing capability; therefore, clinical methods are necessary to relieve the symptoms and repair the damaged tissue. However, the current clinical techniques result in inferior repair tissue compared to native articular cartilage and therefore fail to provide a long-term solution [3,4]. Mesenchymal stem cell (MSC) based tissue engineering is viewed as a promising method for long-term repair of cartilage lesions. In tissue engineering, the MSCs are combined with scaffolds and chondrogenic signaling molecules to produce cartilage constructs [5–12]. Despite these advances, tissue-engineered cartilage constructs display inferior mechanical strength compared to native cartilage. Due to the load bearing nature of articular cartilage, engineered cartilage constructs must display higher mechanical strength, which the extracellular matrix (ECM) of the constructs provides. The ECM of adult human articular cartilage contains highly organized collagen fibrils that vary with tissue depth [13]. Mathematical modeling has indicated that the depth-dependent ECM structure of articular cartilage enhances the mechanical properties of the tissue [14,15]. Unlike native articular cartilage, however, tissue engineered cartilage constructs lack this oriented microstructure and therefore display lower mechanical properties. Methods to control the ECM structure of engineered cartilage constructs can help overcome this limitation. Microfluidic gradient generators have been useful to study several biological processes such as pro- and eukaryotic cell chemotaxis [16,17] and neural stem cell proliferation and differentiation [18]. These devices can be fabricated by photo- and soft-lithography methods, made of poly(dimethyl siloxane) (PDMS), and are amenable to cell culture. This study investigated the use of a gradient generating microfluidic device to study the effects of fluid flow-induced shear stress on MSC alignment and biomolecular gradients on MSC chondrogenesis. In a previous study, aligned MSCs undergoing chondrogenesis synthesized aligned type II collagen and resulted in tissue constructs with enhanced mechanical properties [19]. Previous studies have also indicated that exposure of cells, including endothelial cells and MSCs, to fluid flow induced shear stress results in cellular alignment along the flow direction [20–22]. Since the gradient-generating device used in this study develops gradients via fluid flow, it can be used to study the effects of shear stress on MSC alignment, which may be utilized to control ECM structure. The exposure of MSCs to chondrogenic biomolecular gradients may serve as another method to control tissue structure. During human development, biochemical gradients serve as environmental signals to produce organized tissues by controlling cell migration and differentiation [23,24]. In this study, microfluidic devices created via PDMS soft-lithography were used to subject human MSC monolayers to fluid flow induced shear stress and to transforming growth factor β1 (TGF-β1) gradients. Our results show that when the cells were both confluent and exposed to shear stress, they aligned along the flow direction. Further, exposure to TGF-β1 gradients led to chondrogenesis. These results, together with a previous study that showed that aligned MSCs produce aligned collagen, suggest that oriented cartilage tissue structures with superior mechanical properties can be obtained by using flow and chondrogenic factor gradients formed by microfluidics [19].

## 2. Materials and Methods

### 2.1. Materials

Dulbecco’s Modified Eagle’s Medium Low Glucose (DMEM-LG) (1.5 g/L), Dulbecco’s Modified Eagle’s Medium High Glucose (DMEM-HG) (4.5 g/L), fetal bovine serum (FBS), trypsin-EDTA, bovine serum, sodium pyruvate, human plasma fibronectin, antibiotic-antimycotic cocktail, and phosphate buffered saline (PBS) were obtained from Invitrogen (Carlsbad, CA, USA). Dexamethasone, *N*-propyl gallate, glycerol, and pronase was purchased from Sigma Chemical Co. (St. Louis, MO, USA). SU-8 2075 negative photoresist, SU-8 developer, and buffered oxide etch were obtained from MicroChem Corp. (Westborough, MA, USA). Mylar photomasks were obtained from Advanced Reproductions Corporation (North Andover, MA, USA). Sylgard 184 elastomer base and Sylgard curing agent were obtained from Dow Corning Corporation (Midland, MI, USA). Disposable tissue culture plastics were obtained from Fisher Scientific (Waltham, MA, USA). Leur lock connectors were obtained from McMaster-Carr (Aurora, OH, USA). Platinum cured silicone tubing was obtained from Cole Parmer (Vernon Hills, IL, USA). ITS+ premix tissue culture supplement was obtained from Becton Dickinson (Franklin Lanes, NJ, USA). (Tridecafluoro-1, 1,2,2-tetrahydrooctyl)-1-trichlorosilane was purchased from UCT Specialties (Bristol, PA, USA). Fibroblast growth factor-2 (FGF-2) and TGF-β1 were purchased from Peprotech (Rocky Hill, NJ, USA). Ascorbate-2phosphate (A2P) was obtained from Wako (Richmond, VA, USA). Fluorescein isothiocyanate (FITC)-conjugated goat anti-mouse IgG secondary antibody was obtained from MP Biomedicals (Irvine, CA, USA). The type II collagen primary antibody was purchased from the Developmental Studies Hybridoma Bank (University of Iowa, Iowa City, IA, USA).

### 2.2. Design of Microfluidic Gradient Generating Device

**Figure 1.**
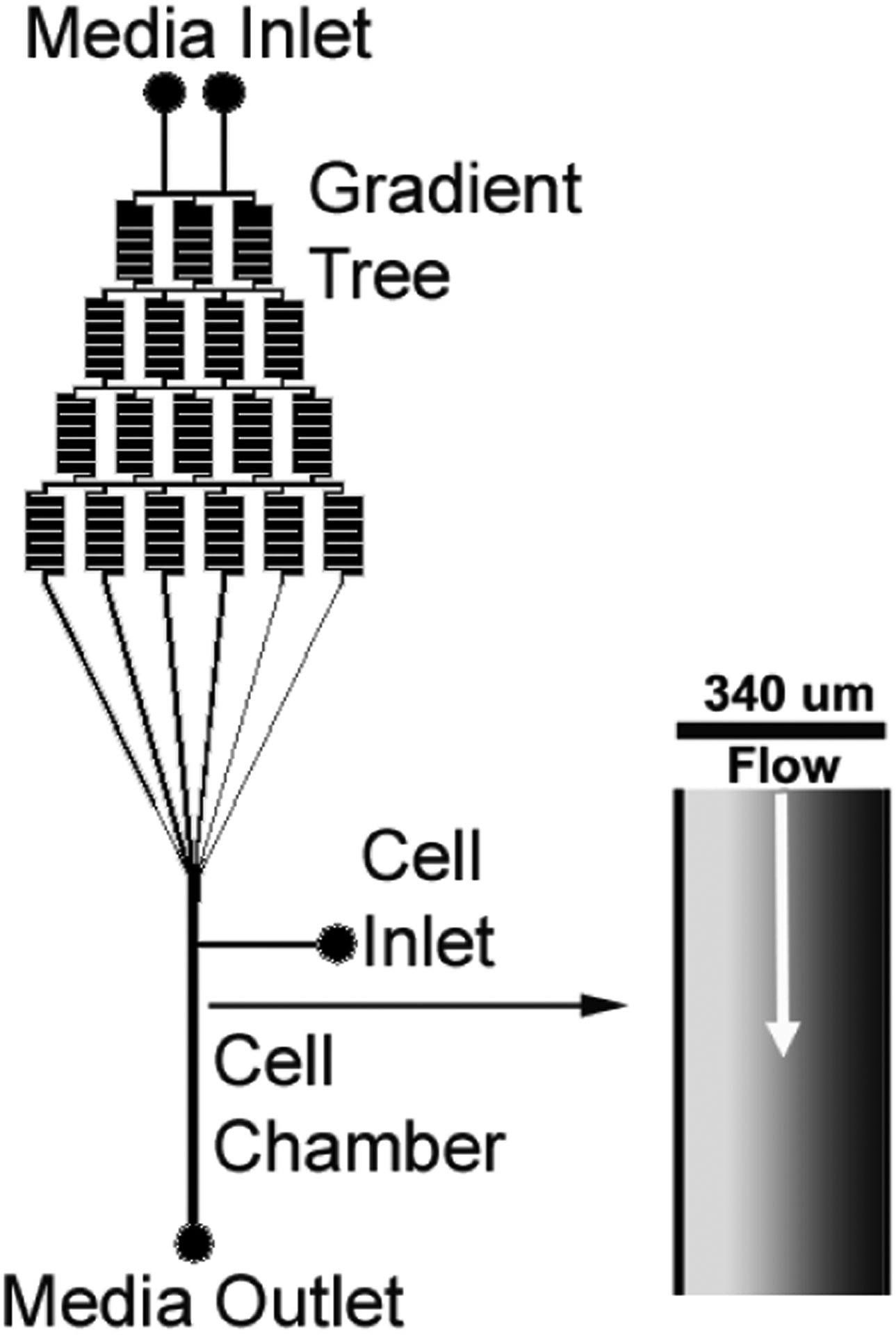
Microfluidic gradient generating chamber design.

A microfluidic gradient generating design characterized by Jeon *et al.*[17] was adapted and produced in AutoCAD (Autodesk, Inc., San Rafael, CA, USA). The base microfluidic gradient generating design was developed by Dertinger *et al.* [25]. The design (Figure 1) utilizes two flow inlets that can contain cell medium of different molecular compositions. Mixing is driven by diffusion and convection within the gradient generating tree. This gradient generating tree ends at a chamber, producing a molecular gradient across the chamber width. An inlet next to the chamber allows for easy cell seeding within the device. A single outlet is present at the end of the cell observation chamber. The chamber width was 340 μm, and the length was 15 mm. For the MSC alignment experiments, the chamber depth was 140 μm. For the MSC chondrogenesis experiments, the chamber depth was 70 μm. The chamber volumes were 0.714 and 0.357 μL respectively. The chamber surface area (bottom) was 5.1 mm^2^. Standard microfabrication techniques were used to produce the gradient generating design on a silicon substrate using SU-8 2075 negative photoresist [26]. Mylar photomasks containing the design were used during the microfabrication process, which was performed within the Electronics Design Center at the Case Western Reserve University. A total of 4 devices were run for cellular alignment experiments and 2 devices were run for MSC chondrogenesis experiments.

### 2.3. PDMS Soft Lithography

PDMS soft lithography, as described by Xia and Whitesides [27,28], was used to produce the gradient generating patterns within PDMS. Briefly, Sylgard^®^ 184 elastomer base was mixed 10:1 with the curing agent, and the mixture was poured over silicon wafers with the negative photoresist patterns. After degassing under vacuum, the mixture was cured at 80 °C overnight. To form a device, the cured PDMS membranes were bonded to a glass slide after exposure to oxygen plasma (Plasma Prep^™^ II SPI Supplies, West Chester, PA, USA). Prior to bonding, holes were punched for the inlets and outlet of the microfluidic device. Silicone tubing was attached to the ports along with Leur-lock connectors to the inlet tubing and a waste collection 50 mL tube to the outlet. The entire device was then sterilized using an autoclave at 121 °C for 20 min.

### 2.4. MSC Isolation and Expansion

Human MSCs were isolated from bone marrow aspirates obtained from healthy donors through the Stem Cell Core Facility of the Case Comprehensive Cancer Center. Donors provided informed consent under terms of an IRB-approved protocol prior to the harvesting of the aspirates. The isolation of the MSCs was completed by trained staff within the Skeletal Research Center at Case Western Reserve University. The MSCs were isolated based on their ability to attach to cell culture plastic and proliferate under specific cell culture conditions as described by Lennon *et al.* [29].

Expansion of MSCs was conducted using MSC growth medium: DMEM-LG with 10% FBS and 10 ng/mL FGF-2. The FBS was selected as described by Lennon *et al.* [29] to maintain the MSCs in their undifferentiated state during expansion. The addition of FGF-2 has been shown to enhance the chondrogenic potential of MSCs [30,31]. After expansion, the MSCs were frozen at passage 1. The MSCs were thawed and then cultured to 80%–90% confluence, with growth medium changes every 2–3 days. Once 80%–90% confluence was reached, the MSCs were subcultured and were resuspended in MSC growth medium for experimental use at passage 2. A single donor was used for all the cellular alignment experiments. Another single donor was used for the chondrogenic experiments.

### 2.5. Microfluidic Device MSC Seeding and Culture

**Figure 2.**
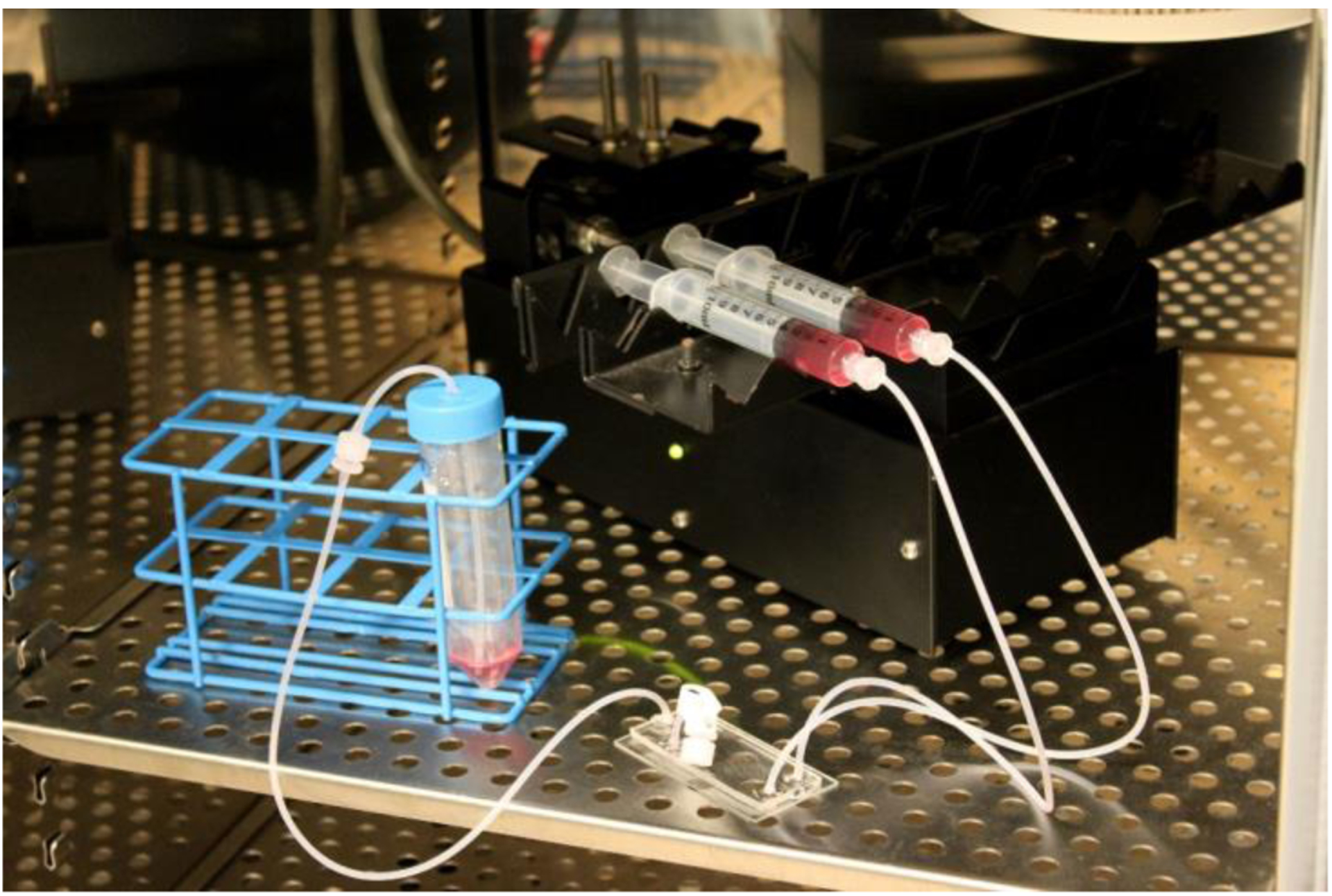
Gradient generating device MSC culture set-up.

Under sterile conditions, the microfluidic device was flushed with 100% ethanol through all 3 inlets to wet the flow channels and to remove air bubbles. To remove the ethanol, the device was flushed with PBS twice. To facilitate cellular attachment to the glass bottom of the flow channels, 20 μg/mL human plasma fibronectin in PBS was injected into the device, which was then incubated at 37 °C for 1 h to allow attachment. Next, MSCs were trypsinized and resuspended in MSC growth medium. The microfluidic device was then flushed with growth medium to remove the fibronectin solution, and the MSC suspension was added through the cell inlet. The tubing of the device was then clamped to keep the cell suspension within the cell observation chamber, and the device was stored in a humidified environment at 37 °C and 5% CO_2_ for 3 h to allow for cell attachment. After cell attachment, syringes with growth medium were attached to the device and were placed on a Harvard Apparatus syringe pump (PHD 2000 series, Holliston, MA, USA). The entire set-up was placed in a 5% CO_2_ containing humidified air environment at 37 °C, and flow was initiated using the syringe pump (Figure 2). Static controls (0 dynes/cm^2^) for shear stress experiments consisted of MSCs seeded within a petri dish.

### 2.6. Alignment and Shear Stress Characterization

The MSCs were cultured under a flow of growth medium at different flow rates resulting in the following shear stresses: 0.1, 0.12, 0.2, and 0.4 dynes/cm^2^. The corresponding inlet fluid flow rates were 25, 30, 50, and 100 μL/h respectively. Static controls (0 dynes/cm^2^) consisted of MSCs seeded within cell culture treated polystyrene petri dishes (no fibronectin treatment). Shear stress was calculated based on the following equation, where *d* is the depth (140 μm for alignment experiments, 70 μm for chondrogenic gradient experiments) and *W* is the width of the cell observation channel (340 μm), *Q* is the cell observation channel volumetric flow rate, and *u* is the fluid (cell culture medium) viscosity.

(1) $$\tau =\frac{6u\text{Q}}{d^{2}W}$$

For the lowest shear stress, 0.1 dynes/cm^2^, MSCs were seeded at a confluent state and alignment was observed over 4 days. For the remaining shear stress values, MSCs were seeded at a non-confluent state and alignment was quantified over 8 days. For these devices, phase contrast images were taken after MSC attachment (just prior to the start of flow, day 1) and on days 3, 6, and 8 using an Olympus IX71 inverted microscope (Olympus, Tokyo, Japan) with UPlanFl 10× and LCPlanFl 20× objectives. To determine the effect of shear stress on MSC alignment, the acute angle of the long axis of individual cells relative to the flow direction was measured using ImagePro software (Media Cybernetics, Inc., Bethesda, MD, USA). An angle of 0° indicates cellular alignment parallel to flow, while an angle of 90° indicates cellular alignment perpendicular to flow. Using Microsoft Excel (Redmond, WA, USA), the average alignment angles were plotted at different time points. Alignment angles were also plotted in frequency histograms and grouped in 15° angle bins. Differences in alignment were observed in confluent *vs.* non-confluent regions of the device; therefore, these regions were analyzed and plotted separately to determine the effect of cell confluence on alignment. When the monolayer of MSCs had no open spaces, the region was considered confluent. Using Origin 9.1 (Origin Lab, Northampton, MA, USA), box plots of the cell alignment angles were created for select conditions and time points.

### 2.7. MSC Chondrogenic Differentiation

For the chondrogenic experiments, the growth medium syringes were changed to chondrogenic medium after the MSCs reached confluency within the device. Cell culture medium to initiate chondrogenesis of MSCs was initially developed by Yoo *et al.* and consisted of the following: DMEM-HG supplemented with 1% ITS+ premix tissue culture supplement, 10^−7^ M dexamethasone, 37.5 μg/mL A2P, 1% sodium pyruvate, and 10 ng/mL TGF-β1 [32]. A syringe containing chondrogenic medium with 10 ng/mL TGF-β1 was attached to one inlet and a syringe containing chondrogenic medium without TGF-β1 was attached to the other inlet. Flow was then initiated resulting in a linear gradient of TGF-β1 across the channel width [17]. For the constant concentration chondrogenic control, syringes with chondrogenic medium containing 10 ng/mL TGF-β1 were attached to both inlets. The devices were cultured for 21 days under an inlet flow rate of 100 μL/h (1.7 dynes/cm^2^ shear stress), and phase contrast images of the cell observation chamber were taken at various time points during the 21 day time period. One gradient and one control device were used for the chondrogenic experiments.

### 2.8. MSC Aggregate Analysis

After 21 days in chondrogenic culture, phase contrast images were taken of the MSC aggregates and were used for analysis. The total number of aggregates were counted in both the gradient and control device. Only aggregates that had a long axis of at least 100 μm were counted. Using ImageJ software (National Institutes of Health, Bethesda, MD, USA), regions of interest were traced around each aggregate. The projected area of each aggregate (region of interest) was then measured. An ellipse was then fit to each of the aggregates. Using the major and minor axis of the ellipse, the aspect ratio of the aggregates was calculated. The angle of the major axis of the best fit ellipse was measured with respect to the flow direction to determine the aggregate alignment angle with respect to flow. The aggregate areas, aspect ratios, and alignment angles were plotted as box plots.

### 2.9. Type II Collagen Immunohistochemistry

After 21 days in chondrogenic culture, immunohistochemistry (IHC) was used to detect the presence of type II collagen within the aggregates. IHC was performed via fluid flow of the following components into the device. Antigen unmasking was performed via flow of 1 mg/mL pronase in PBS for 15 min at room temperature. After washing the aggregates with PBS for 10 min, they were then blocked with 10% normal goat serum (NGS) in PBS for 30 min. The type II collagen primary antibody, diluted 1:50 in 1% NGS, was applied to the aggregates for 60 min using flow. The primary antibody host species is mouse and the antigen species is chicken. It has been shown to have cross-reactivity with human type II collagen [19,31] synthesized by human MSCs. The samples were then washed in PBS for 1 h. FITC-conjugated goat anti-mouse IgG, diluted 1:500 in 1% NGS, was then applied to the aggregates for 45 min. The samples were again washed with PBS for 1 h. The samples were then wet mounted by using 5% *N*-propyl gallate in glycerol. The fluorescent images were captured. Secondary antibody only controls were not completed because we performed the collagen type II staining in-situ on the entire sample and subsequently, imaged the sample while it was still in the device. Washing steps were performed in a convective environment and expected to yield better results than static conditions (sections mounted on slides). Our previous experience with sections from MSC based cartilage constructs indicate that non-specific staining is not an issue

### 2.10. Statistical Analysis

Statistical analysis was performed using Minitab 17 statistical software (Minitab Inc., State College, PA, USA). Pairwise comparisons were made using the Mann-Whitney test with a *p* value of less than 0.05 considered statistically significant. Sample sizes are indicated in the figure legends. Error bars represent standard error of the mean (SEM).

## 3. Results

### 3.1. Effect of Shear Stress on MSC Alignment

**Figure 3.**
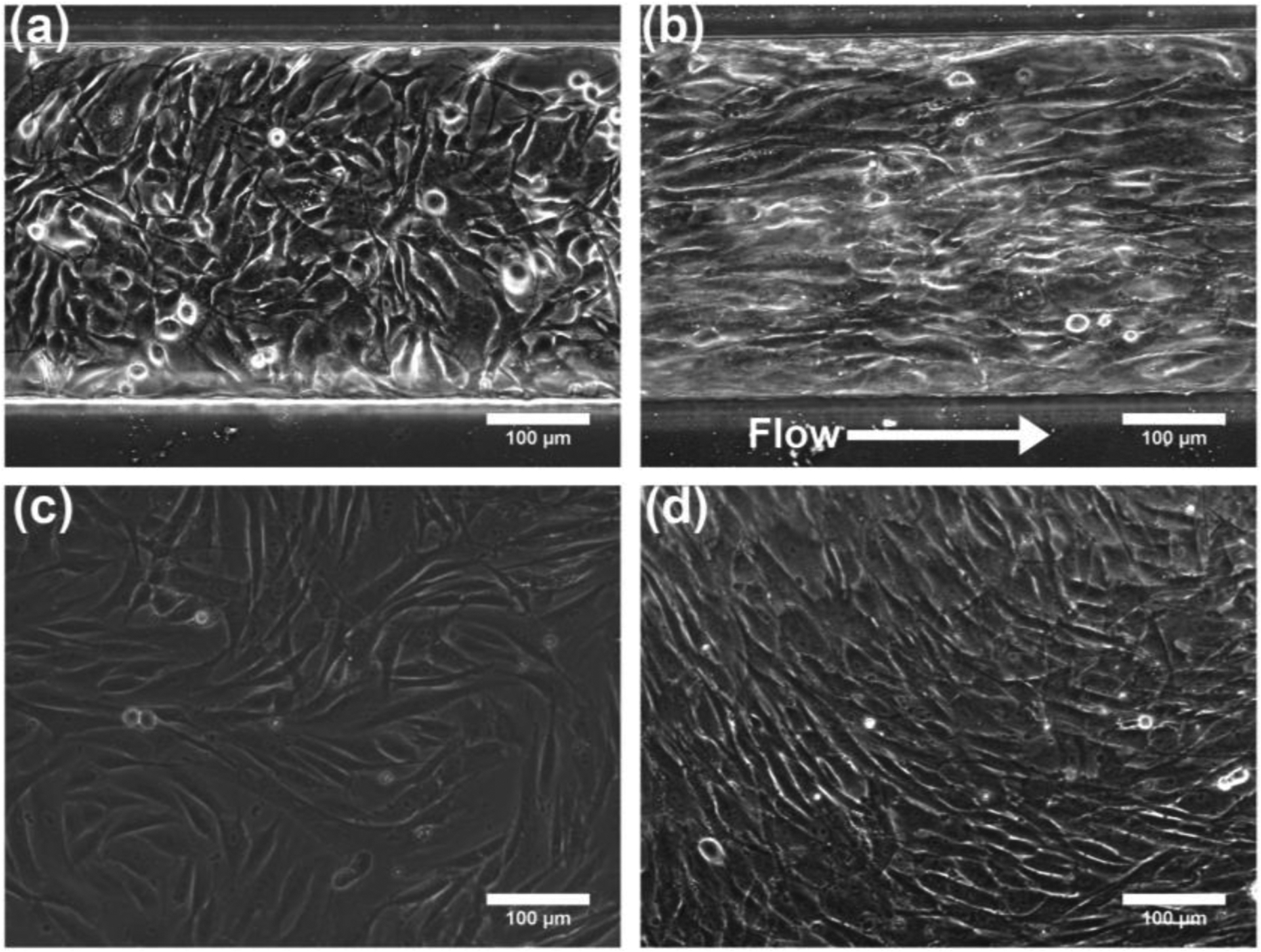
Effect of shear stress on MSC alignment. Phase contrast images of MSCs cultured within the microfluidic device on (**a**) day 1 (just after attachment and before the start of flow) and (**b**) day 4 under a shear stress of 0.1 dynes/cm^2^. Phase contrast images of MSCs cultured in static conditions (0 dynes/cm^2^, polystyrene cell culture treated petri dish) on (**c**) day 1 and (**d**) day 4.

After seeding MSCs within the microfluidic cell observation chamber, the MSCs were cultured within the device until confluency was reached by flowing MSC growth medium throughout the chamber. The effect of shear stress was investigated as the cells proliferated. Specifically, the effect of different flow rates, which resulted in shear stresses ranging from 0.1 to 0.4 dynes/cm^2^, on cellular alignment was observed over time. MSCs were seeded at a confluent state and exposed to a shear stress of 0.1 dynes/cm^2^ (25 μL/h inlet flow rate). By day 4 in microfluidic culture, the long axis of the MSCs aligned preferentially along the flow direction at this shear stress (Figure 3). In comparison, MSCs cultured under static conditions (0 dynes/cm^2^) exhibited no preferential overall cellular alignment. While MSC alignment was observed in small localized regions under static conditions, these regions differed from each other in alignment angle, resulting in no preferential alignment for the overall sample.

**Figure 4.**
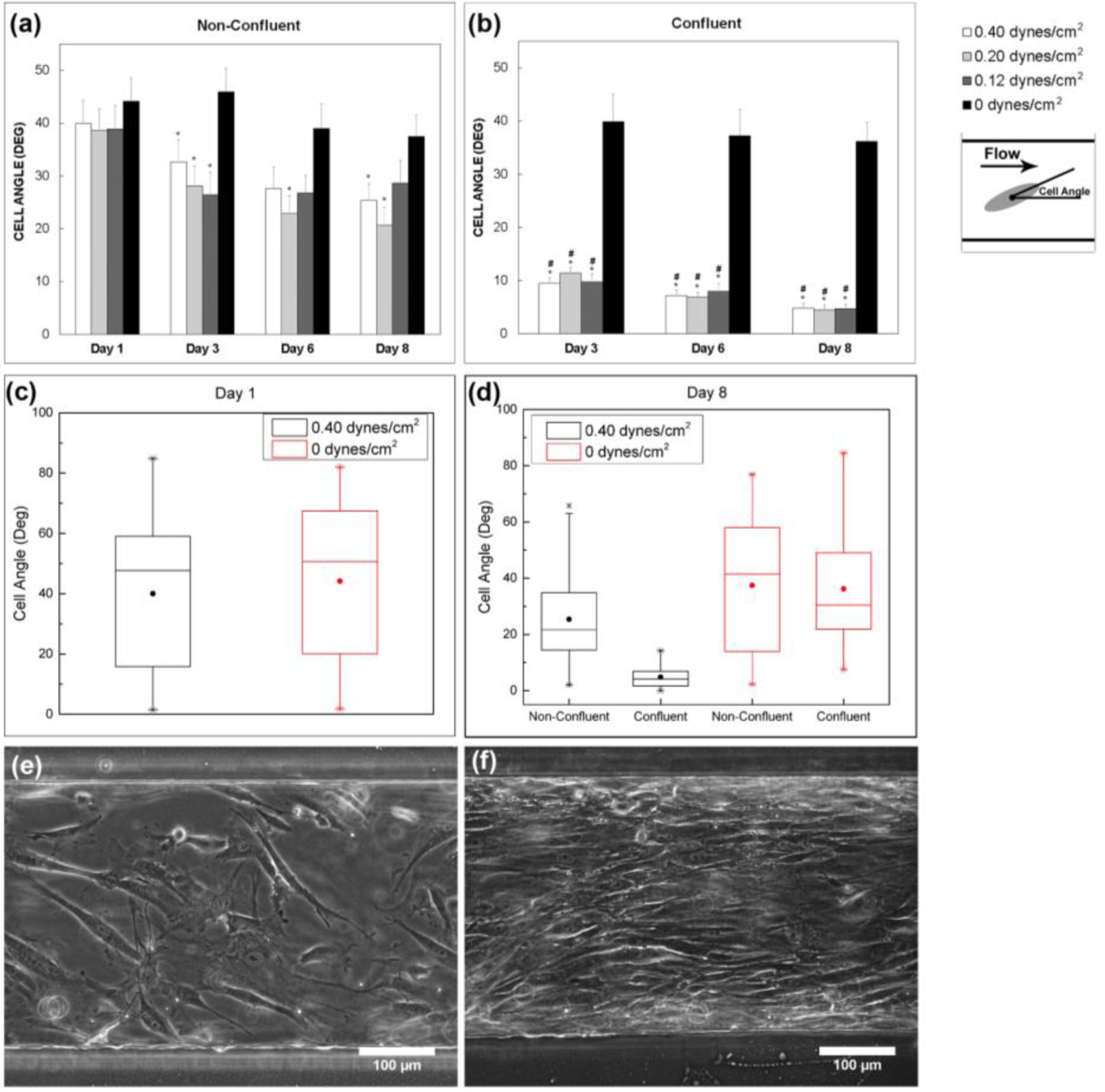
Effect of shear stress on MSC alignment (average cell angle). Average cell angle was plotted at different time points and shear stress values for (**a**) non-confluent regions and (**b**) confluent regions. Day 1 angle measurements were taken after MSC attachment and just prior to flow. * indicates a statistical difference between the flow based conditions and the static control. # indicates a statistical difference between the confluent and non-confluent angle measurements for a given shear stress condition and time point (*p* < 0.001). MSCs were seeded at a non-confluent state, so no confluent regions were present on day 1. Cell angles for the 0.40 and 0 dynes/cm^2^ conditions were also plotted as box plots for (**c**) day 1 and (**d**) day 8. The lower and upper boundaries of the box represent the first and third quartiles respectively, while the line within the box represents the median of the data. The whiskers represent the minimum and maximum data points not including outliers. The solid circle indicates the mean value and * indicates the minimum and maximum data points including outliers. (**e**) Representative non-confluent region of the 0.40 dynes/cm^2^ device on day 3. (**f**) Representative confluent region of the 0.40 dyes/cm^2^ device on day 3. *N* = 30 cells were measured per shear stress for each time point and cell density. The static controls (0 dynes/cm^2^) were cultured on polystyrene cell culture treated petri dishes. Error bars represent SEM.

**Figure 5.**
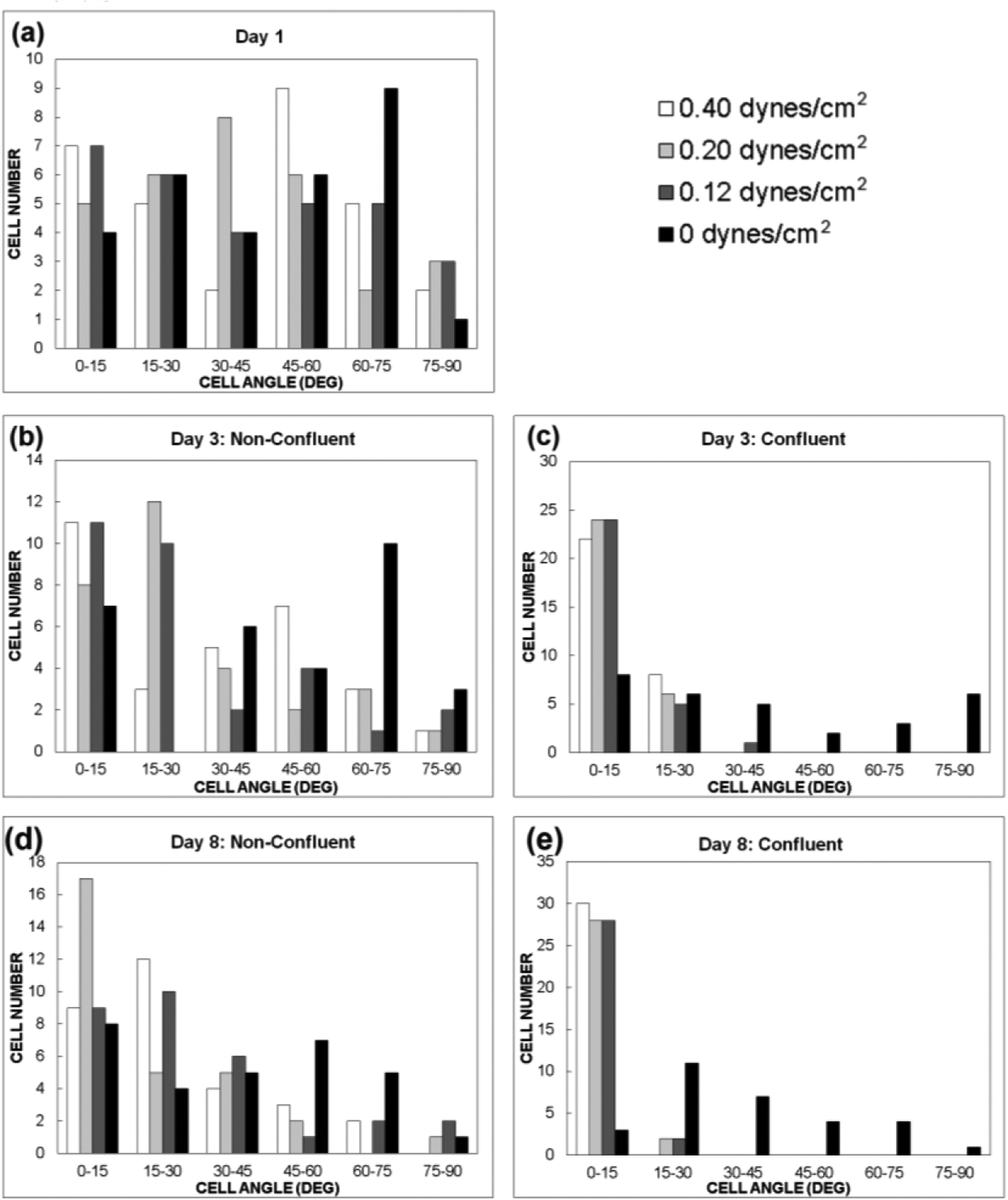
Effect of shear stress on cell alignment (Cell angle distribution). The number of cells that displayed angles within the designated ranges were plotted for (**a**) day 1 (after MSC attachment, prior to fluid flow), (**b**) non-confluent regions on day 3, (**c**) confluent regions on day 3, (**d**) non-confluent regions on day 8, and (**e**) confluent regions on day 8. MSCs were seeded at a non-confluent state, so no confluent regions were present on day 1. The static controls (0 dynes/cm^2^) were cultured on polystyrene cell culture treated petri dishes. *N* = 30 cells were measured per shear stress condition for each time point and cell density.

Over an 8-day time period, cellular alignment was quantified by measuring the acute angle of the long axis of the cells with respect to the flow direction for 3 different shear stress values: 0.12, 0.2, and 0.4 dynes/cm^2^. The MSCs were seeded in a non-confluent state on day 1 and then exposed to inlet flow rates of 30, 50, and 100 μL/h. An angle of 0° indicates cellular alignment along the flow direction. Under flow conditions, regions of the microfluidic device that were confluent displayed enhanced alignment along the flow direction when compared to non-confluent regions (*p* < 0.001), indicating that both shear stress and physical guidance via cell to cell contact are necessary to align the MSCs. The average cell angle was measured for different shear stresses at different time points (Figure 4). In addition, the number of cells within a specified angle range were measured for each shear stress condition over different time points (Figure 5). The confluent regions were measured separately from the non-confluent regions. By day 3, the cells exposed to shear stress within confluent regions were mostly aligned along the direction of flow. In contrast, cells exposed to shear stress within the non-confluent regions displayed less alignment but still more alignment than the static control. By day 8, nearly all the cells exposed to shear stress within confluent regions displayed an angle between 0 and 15°, indicating alignment along the flow direction. Once again, the non-confluent regions exposed to shear stress exhibited less alignment compared to the confluent regions. Over the 8-day period, in the range of shear stresses tested, there was no difference in their effect on the degree of cellular alignment (*p* > 0.2) for both confluent and non-confluent regions. Thus, the results indicate that the presence of shear is sufficient to induce cellular alignment.

### 3.2. Effect of TGF-β1 Gradients on MSC Chondrogenesis

**Figure 6.**
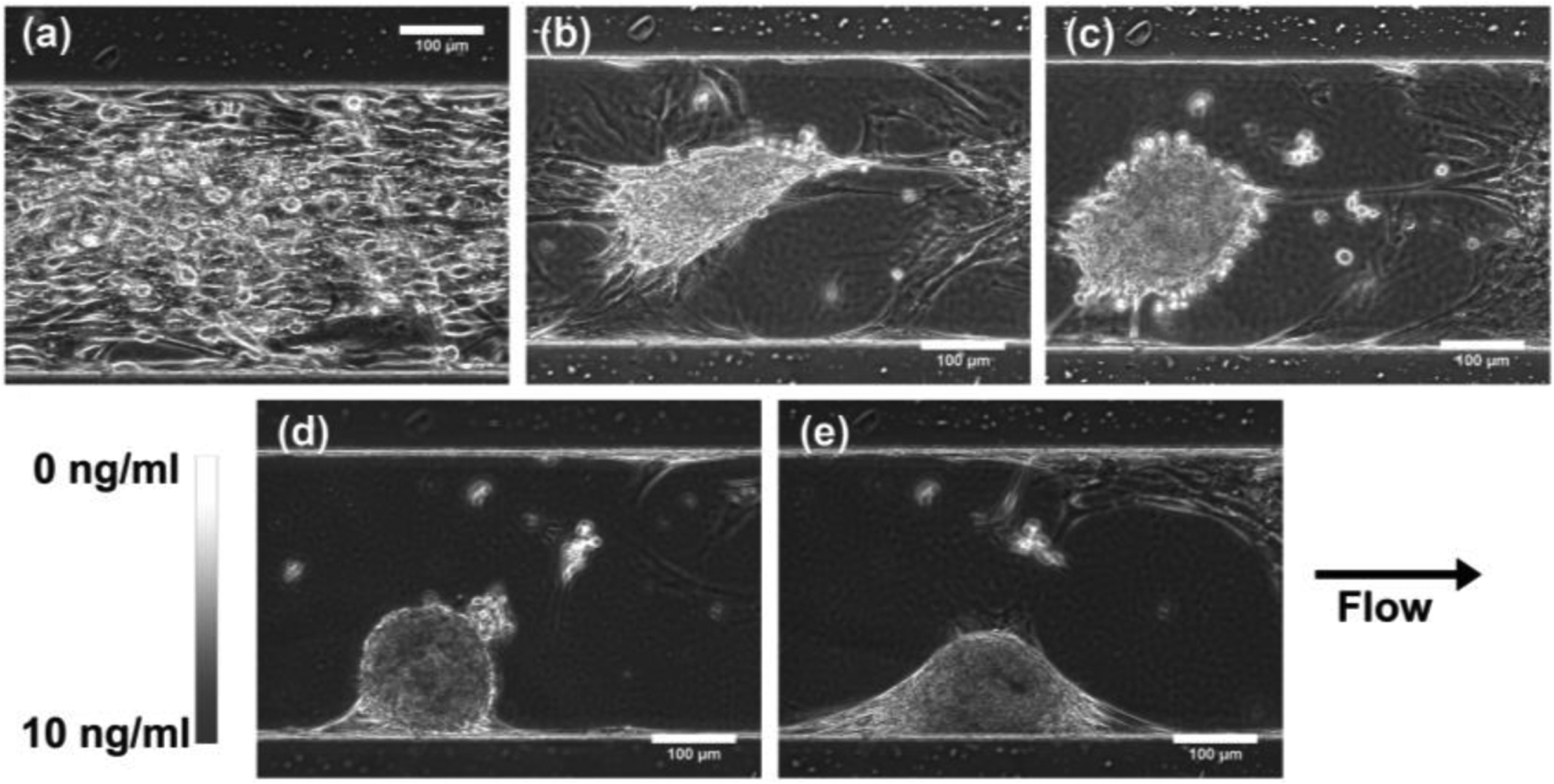
Effect of TGF-β1 gradient on MSC chondrogenesis. Phase contrast images of a region within the gradient device on (**a**) day 1 (just prior to TGF-β1 gradient exposure), (**b**) day 6, (**c**) day 8, (**d**) day 14, and (**e**) day 21.

**Figure 7.**
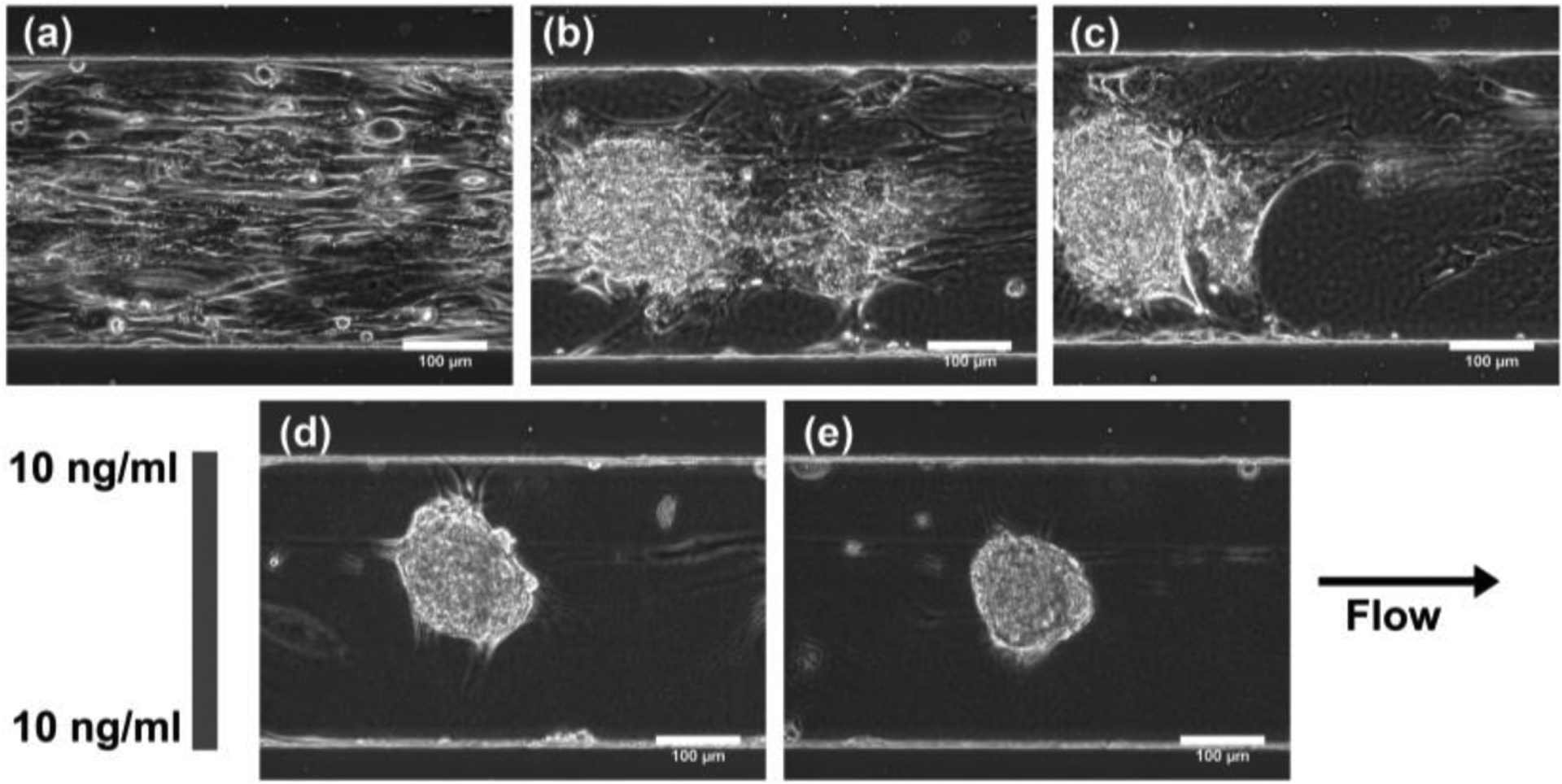
Effect of constant TGF-β1 concentration on MSC chondrogenesis. Phase contrast images of a region within the control device on (**a**) day 1(just prior to TGF-β1 exposure), (**b**) day 6, (**c**) day 8, (**d**) day 14, and (**e**) day 21.

**Figure 8.**
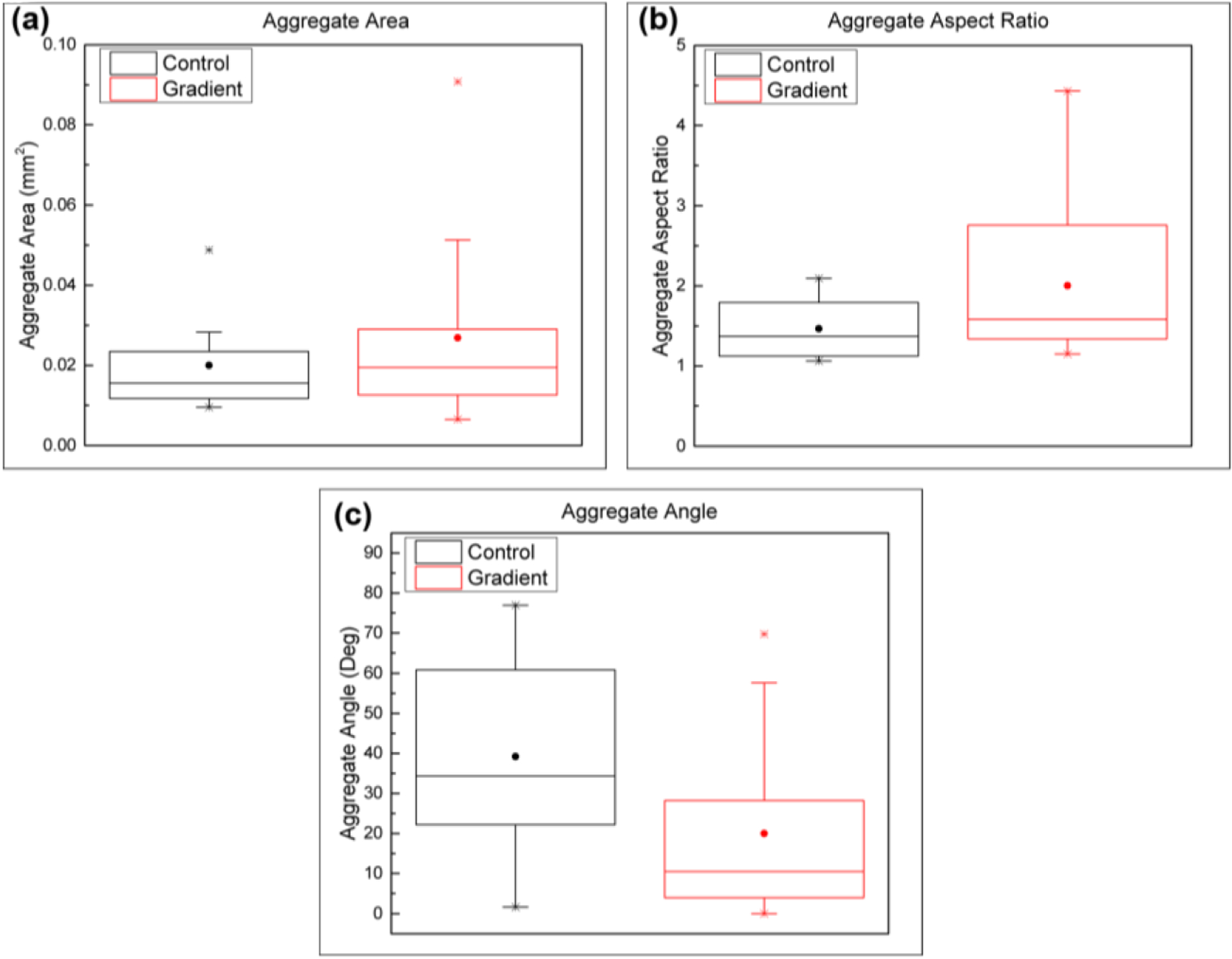
Effect of TGF-β1 gradients on MSC aggregate properties. Box plots of (**a**) aggregate area (*p* = 0.24), (**b**) aggregate aspect ratio (*p* = 0.07), and (**c**) aggregate alignment angle (*p* = 0.04). The alignment angle measurements for the gradient and control devices displayed a statistical difference with a *p* value less than 0.05 using the Mann-Whitney test. The box plot attributes are the same as that in Figure 4. *N* = 14 and *N* = 8 aggregates were used for the gradient and control devices respectively.

**Figure 9.**
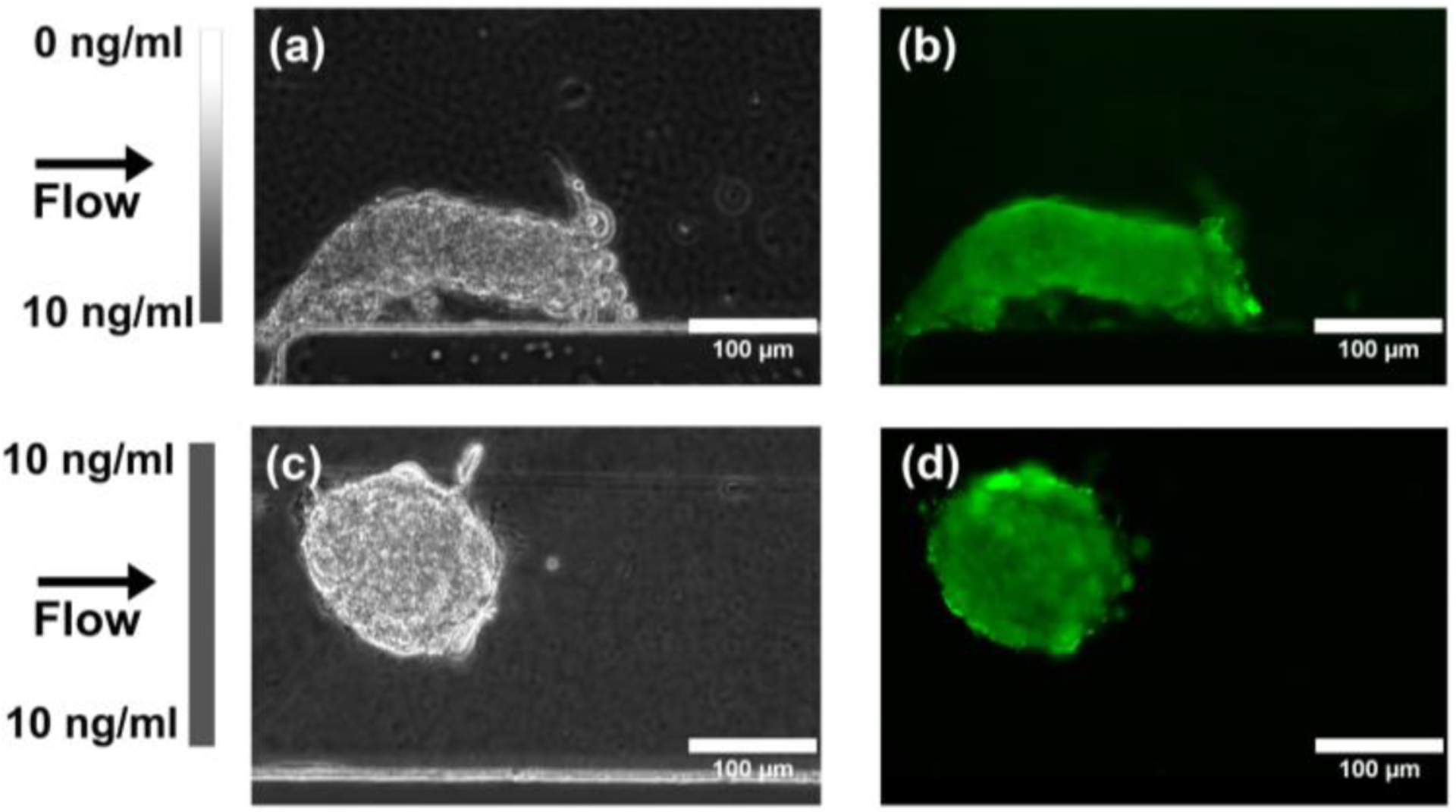
Effect of TGF-β1 gradients on MSC type II collagen deposition (type II collagen IHC). (**a**) Phase contrast image and (**b**) Fluorescent image showing type II collagen deposition within an MSC aggregate after 21 days in TGF-β1 gradient culture. (**c**) Phase contrast image and (**d**) Fluorescent image showing type II collagen deposition within an MSC aggregate after 21 days in constant TGF-β1 concentration culture. Green is type II collagen.

Once the MSCs reached confluency within the microfluidic observation chamber, the MSCs were exposed to a linear gradient of TGF-β1 that ranged from 0 ng/mL to 10 ng/mL across the 340 μm width of the chamber or a constant concentration (10 ng/mL) of TGF-β1 for the control. During the 21-day period, the MSCs exposed to the TGF-β1 gradients started as a monolayer and then gradually contracted together to form chondrogenic aggregates (Figure 6). Compared to the constant concentration control (Figure 7), the TGF-β1 gradient device resulted in more aggregates formed. A total of 14 MSC aggregates formed within the gradient device, while 8 MSC aggregates formed within the control device. The aggregates within the gradient device displayed a higher average projected area, although not statistically different, when compared to the control device (Figure 8). The total aggregate area within the gradient device (0.376 mm^2^) was higher than the total aggregate area within the control device (0.160 mm^2^). In addition, the MSCs exposed to the gradient formed aggregates that were more elongated (higher aspect ratio, not statistically different, *p* = 0.07) and aligned (lower angle, statistically significant, *p* = 0.01) in the flow direction, indicating that gradients of TGF-β1 may be used to control the structure of the chondrogenic aggregates (Figure 8). To assess chondrogenesis within these aggregates, type II collagen IHC was performed after 21 days and indicated that type II collagen, a primary ECM component of articular cartilage, was present in aggregates that formed within the control and gradient devices (Figure 9).

## 4. Discussion

Using the gradient generating design characterized by Jeon *et al.* [17], gradient generating microfluidic devices were fabricated via PDMS soft lithography and used to study the effect of shear stress and TGF-β1 gradients on MSCs. The effect of shear stress on cellular alignment as the MSCs proliferated under flow of MSC growth medium was investigated. In a previous study, we showed that aligned MSCs produce aligned ECM and enhance the mechanical properties of tissue-engineered constructs [19]. Therefore, the ability to align MSCs using flow will allow for control of ECM structure and mechanical properties. Endothelial cells have been known to change orientation and align in the direction of fluid flow [20,21]. Recently, MSCs have also been shown to align when exposed to fluid flow-induced shear stress [22]. However, another study exposing non-confluent monolayers of MSCs to fluid flow based shear stress did not observe changes in MSC morphology in response to shear stress [33]. In our study, when a confluent monolayer of MSCs was exposed to fluid flow-induced shear stress, the cells aligned along the flow direction. Less cellular alignment was observed in non-confluent regions of the microfluidic device, indicating that the cell alignment was influenced by both shear stress and cell to cell contacts present under confluent conditions. In static control samples not exposed to shear stress, no overall cellular alignment was observed although small confluent regions did exhibit localized alignment. However, because these regions were aligned in different directions, no preferential alignment within the overall sample was observed. These results demonstrate that the application of shear stress within this gradient generating device will be a useful method for aligning MSCs with the goal of producing aligned ECM.

Once the cell observation chamber within the microfluidic device was confluent, the MSCs were exposed to a flow-generated TGF-β1 gradient within chondrogenic medium for a period of 21 days. During this period, the aligned MSCs gradually contracted and formed chondrogenic aggregates within the cell observation chamber. Although smaller, these aggregates macroscopically resemble those produced during standard MSC pellet chondrogenesis, in which MSCs are added to a conical polypropylene 96 well plate and aggregate to form pellets under chondrogenic culture conditions [34]. While the aggregates formed within the chamber will affect local gradient profiles, a molecular gradient could still be maintained over the aggregates since the aggregate heights were smaller than the chamber depth. When compared to control MSCs exposed to a constant TGF-β1 concentration, the MSCs exposed to a TGF-β1 gradient formed more aggregates, and the aggregates were more elongated in the flow direction compared to the control device. Type II collagen staining confirmed the synthesis of type II collagen within the aggregates, confirming chondrogenesis of the MSCs within the devices. One explanation for the enhanced elongation of the chondrogenic aggregates exposed to the TGF-β1 gradient involves the ability of TGF-β1 gradients to spatially regulate the contractile forces within the aggregate due to changes in concentration. Chondrogenesis has been shown to occur in a dose dependent manner with respect to TGF-β1 concentration [35,36]. Such gradients may be useful for spatially controlling chondrogenesis and the resulting aggregate structure by exposing the MSC monolayer to different doses of TGF-β1 across the observation chamber. The observation chamber within the device also allows for real-time monitoring of the chondrogenesis and MSC aggregation dynamics in response to the biomolecular gradients. These results establish the capability of this microfluidic device to be used for the study of the effect of biomolecular gradients on MSC chondrogenesis and aggregate formation over time.

Further, this device can be used to study osteochondrogenesis by exposing the MSCs to opposing chondrogenic and osteogenic gradients. The exposure of MSCs to biomolecular gradients is a promising method to develop osteochondral tissues that transition from a cartilage-like region to a bone-like region. Studies exposing MSCs to opposing osteogenic and chondrogenic biomolecular gradients immobilized within the scaffolds have produced tissue constructs that transition from an osteogenic region to a chondrogenic region [37,38]. In addition, the ability of this gradient generating device to simultaneously expose MSCs to shear stress may enhance the osteogenic potential of MSCs during osteochondrogenesis. Previous studies have indicated that shear stresses can upregulate osteogenic gene expression of MSCs [33,39,40]. This gradient generating device is a promising tool to simultaneously expose MSCs to shear stress and biomolecular gradients to investigate MSC osteochondrogenesis.

## 5. Conclusions

Our results demonstrate that the gradient generating microfluidic device can be used to study chondrogenesis of MSCs when exposed to shear stress and biomolecular gradients. Shear stress and TGF-β1 gradients can, in turn, be used to control MSC alignment and chondrogenic aggregate structure respectively. Based on our previous study, aligned MSCs can produce aligned type II collagen and result in tissue constructs with enhanced mechanical properties [19]. This study, together with these previous results, indicates that we can obtain cartilage tissue constructs with oriented ECM and superior mechanical properties by using both shear stress and chondrogenic gradients.
